# CIF extension for *Visual Studio Code*

**DOI:** 10.1107/S1600576725005217

**Published:** 2025-07-08

**Authors:** Kaisa Helttunen, Heikki Kainulainen

**Affiliations:** a Department of Chemistry, Nanoscience Center University of Jyväskylä PO Box 35 Jyväskylä FI-40014 Finland; b Jyväskylä, Finland; Instituto Andaluz de Ciencias de la Tierra, Granada, Spain

**Keywords:** crystallographic information files, CIF dictionary, CIF parser, *Visual Studio Code*

## Abstract

*Visual Studio Code* (*VS Code*) is a popular text editor and integrated development environment commonly used in software development. The crystallographic information file (CIF) language extension makes syntax highlighting, auto completion, error checking and hover information available for crystallographers reading and editing CIFs. The language extension features are available for all files following the CIF syntax, including CIF dictionary files. The design and features of the *VS Code* CIF extension are presented.

## Abstract 2

The design and features of the CIF extension for *Visual Studio Code* are presented.

## 1. Introduction

Since the introduction of the crystallographic information file (CIF; Hall *et al.*, 1991 ▸; Hall & McMahon, 2005 ▸) and the STAR file structure (Hall, 1991 ▸; Hall & Spadaccini, 1994 ▸) it is based on, crystallographers have had a standardized protocol to exchange and archive crystallographic data in electronic databases and journals. The structure of a CIF has relatively few restrictions and it resembles popular data formats such as extensible markup language (XML) in the philosophy of separating form from content (Brown & McMahon, 2002 ▸). The data in a CIF are organized in pairs of data name and data value, that is, as tag–value pairs. Importantly, each data name has to have a value. The data names are collected and described in CIF dictionaries, approved by the International Union of Crystallography (IUCr) committee COMCIFS. Each data name is connected to a specific crystallographic property and maintains its description indefinitely once added to a standard CIF dictionary. CIF syntax also permits the use of non-standard data names, as long as they are new and unique in comparison with the existing dictionaries (IUCr, 2025*a* ▸).

The committee preparing the CIF standard and the authors of the first CIF publication already recognized the significance of visual aids for humans by a recommendation to organize data names of the same category into groups (Hall *et al.*, 1991 ▸). A possibility to edit CIFs manually with a text editor was also mentioned, even though crystallographic programs were expected to generate CIF data items automatically already by the 1990s. In the 30 years since then, crystallographers have benefited greatly from the exponential growth in computing power, from graphical user interfaces and from the development of increasingly user-friendly scientific software.

Computational limitations guided the writing of CIF 1.1 to include restrictions typical of its time. CIF 2.0 and STAR 2.0 were published in 2016, bringing expansion to the character repertoire from ASCII to Unicode, expanded possibilities to present quoted data values, and development of new compound data types, lists and tables (Bernstein *et al.*, 2016 ▸). In CIF 1.1, tabular data are only presented in loops that start with the reserved word loop_ followed by a list of data names and sequences of data values, each containing the same number of values as there are data names in the loop. Current crystallographic software still relies mainly on CIF 1.1, and it is the established format for sharing crystal structure data at the moment. In contrast, recent versions of the CIF dictionaries have been written in CIF 2.0 syntax using DDLm (Spadaccini & Hall, 2012 ▸).

Even though the majority of the content of a CIF is generated automatically by crystallographic software during structure refinement, CIFs are sometimes edited manually. Such cases appear at the final stages prior to data deposition with structural databases, such as the Cambridge Structural Database (https://www.ccdc.cam.ac.uk/products/csd/; Groom & Allen, 2014 ▸; Groom *et al.*, 2016 ▸), the Inorganic Crystal Structure Database (https://icsd.products.fiz-karlsruhe.de; Belsky *et al.*, 2002 ▸) and the Crystallography Open Database (https://www.crystallography.net/; Gražulis *et al.*, 2012 ▸), and submission to scientific journals. Opening CIFs in a text editor is fast for making small corrections, checking formatting, and fixing errors in syntax or crystallographic information, and for viewing details in CIFs published by other laboratories. The files can be very long, since a STAR file has no limitations on the number of data items (Hall, 1991 ▸; Hall & Spadaccini, 1994 ▸). Typically, a CIF contains tens of thousands or hundreds of thousands of lines when the .hkl and the .res file contents have been saved in the CIF for multiple data blocks. Thus, an advanced text editor facilitates all manual editing and viewing.

Over the years, crystallographers have had many advanced programs made available, such as *CIFEDIT* (Toby, 2003 ▸), *enCIFer* (Allen *et al.*, 2004 ▸) and *publCIF* (Westrip, 2010 ▸), providing viewing and editing assistance for CIFs. For example, *enCIFer*, developed by the Cambridge Crystallographic Data Centre, provides both a CIF text editor with syntax highlighting and a structure visualizer. The software provides validation for syntax errors and wizards for inserting bibliographic information and chemical and physical data for the investigated compound. *publCIF* is a CIF text editor available for Windows, Mac and Linux operating systems. It displays the contents of a CIF in two windows: a CIF window and a preprint window, which shows selected sections of crystallographic information in text and tabular formats. The *checkCIF* service offered by the IUCr (2025*b* ▸) is extremely useful for crystallographers, and its properties cover both syntax error checking and an evaluation of whether physically meaningful values have been reported for cell and geometry details, space-group symmetry, anisotropic displacement parameters and structure factors. Overall, *checkCIF* ensures the integrity of crystallographic data in the CIF format.

More modern general purpose text editors offer valuable tools for file editing. *Visual Studio Code* (*VS Code*) is a popular text editor among software engineers (Microsoft, 2024*a* ▸). *VS Code* has important features as a code editor, showing line numbers, syntax highlighting, and a code outline (minimap) for quick navigation and as a visual cue of the current window position relative to the whole file (Microsoft, 2024*a* ▸). *VS Code* can be customized by loading language extensions that the editor automatically suggests according to the file type. With extensions, advanced language-specific features such as auto completion, error checking, hover information and jump to definition are available, which facilitate the work of software developers (Microsoft, 2024*b* ▸). Since CIF is a domain-specific markup language of crystallographic information, crystallographers can also benefit from similar advanced features in a text editor. In this article, we introduce a CIF extension to *VS Code*.

## 2. Methods

The *VS Code* CIF extension was written with TypeScript, which is a superset of JavaScript where static checking for type errors has been added (Microsoft, 2025 ▸). TypeScript code is transpiled into JavaScript before execution. JavaScript allows dynamic properties on webpages, for which it was originally developed as a scripting language. However, it has grown to be a very popular cross-platform programming language for all scales of frontend and backend applications.

The *VS Code* editor offers support for different programming languages through language extensions, which allow advanced editing options such as auto completion and error checking in the code editor. To implement these features in the *VS Code* CIF extension, it is necessary to parse (Aho *et al.*, 2007 ▸; Nystrom, 2021 ▸) the CIF to extract the relevant data. Writing a lexer and a parser for a domain-specific language (DSL), a language like CIF that is focused on a particular domain, is usually relatively simple because DSLs have limited expressiveness and simple syntax in comparison with general purpose programming languages (Fowler, 2011 ▸). The lexer and parser can be written using regular expressions for lexing and a recursive descent parser for syntactic analysis. Another option is to use lexical analysis generators for lexing, parser generators for parsing, or a tool such as *ANTLR* (Parr, 2013 ▸) that combines the lexing and parsing steps more tightly. However, avoiding parser generators may be justified, since they complicate the build process with additional languages and tools (Fowler, 2011 ▸).

In the *VS Code* CIF extension, the lexer is based on regular expressions using the *Regex Table Lexer* design pattern (Fowler, 2011 ▸, ch. 20). During lexing, all text in the CIF is broken down into smaller parts called tokens. In the case of the *VS Code* CIF extension, each token also knows its location relative to the original CIF in the range attribute, which is needed, for example, to place the hover information in the right position in *VS Code*. For example, the first regular expression in Table 1 ▸ recognizes CIF data names because they start with an underscore and end with a whitespace. The first version of the *VS Code* CIF extension supported only CIF 1.1. Recently, we developed limited CIF 2.0 support to include more CIF dictionaries in the software, and the differences in regular expressions between these versions are shown in Table 1 ▸.

The parser of the *VS Code* CIF extension takes the lexer output stream of tokens as input and uses the grammar of CIF explicitly to build a parse tree, where dependencies between the tokens are established. The parser operates as a recursive descent parser (Fowler, 2011 ▸, ch. 21). The parser inspects which data block, data name or loop each token belongs to and connects each data value to the correct data name. After the lexer and parser have analysed the CIF, the *VS Code* CIF extension has information about the content of the CIF, enabling development of smart language editing properties for *VS Code*.

The *VS Code* language extensions provide declarative and programmatic smart editing features (Microsoft, 2024*b* ▸). The *VS Code* CIF extension delivers syntax highlighting, which is a declarative language feature, and hover information, auto completion and error checking from the programmatic language features. The syntax highlighting recognizes keywords, comments, text strings, constants and variables from the source code file and defines how these fragments are displayed in the *VS Code* editor by editing their font colour (Microsoft, 2024*c* ▸). The syntax highlighting involves tokenization and theming, which associates a token with a scope and maps the scopes (tokens) to specific colours. *VS Code*’s tokenization engine utilizes the *TextMate* grammars, a structured collection of regular expressions which were originally developed for the *TextMate* editor (MacroMates, 2024 ▸). Regular expressions are also used here (Table 2 ▸), and they resemble those used in the lexer but are defined independently.

The data name is associated with the variable.other.cif*Textmate* scope, comment text with comment.line.number-sign.cif, data block names with entity.name.function.cif, loops with the keyword.control.cif*Textmate* scope and so on. The *VS Code* editor has several colour themes that the user can choose, and the colour for each of the scopes varies depending on the chosen theme, such as dark or light themes. For example, the data name colour follows the colour reserved for variables in each theme. Therefore, meaningful colours for the syntax highlighting are obtained without defining exact colours in the code of the *VS Code* CIF extension. To demonstrate the potential to adapt the *VS Code* CIF extension to other code editors, a preliminary test of the syntax highlighting with *TextMate* grammars was carried out on *PyCharm* (JetBrains, 2025*b* ▸).

The programmatic language features of the *VS Code* CIF extension (hover information, auto completion and error checking) are implemented via a *Language Server* that utilizes the parse tree. The *Language Server* is a module in *VS Code* and should not be confused with, for example, physical computer servers. The hover information reveals connections between tokens, since this information was investigated during parsing. The range attribute of the token informs the code editor to place the hover in the correct position.

Auto completion and error checking are based on the CIF dictionaries. The current version of the *VS Code* CIF extension contains the COMCIFS approved dictionaries that have been read from the IUCr website register file (IUCr, 2025*c* ▸). The validator has information on the standard data names, their description and their allowed values according to the dictionaries.

The *VS Code* CIF extension complies with the Language Server Protocol (LSP; Microsoft, 2024*d* ▸) which standardizes communication between a *Language Server* and a code editor (Microsoft, 2024*e* ▸). This has several benefits as the *Language Server* can be written with any language, in this case TypeScript, and it is run in a separate process allowing higher performance of the code editor. The LSP was originally developed for *VS Code*, but it has been made an open standard that is widely supported by code editors and integrated development environments (IDEs) such as *Eclipse* (Eclipse Foundation AISBL, 2025 ▸), *Emacs* (LSP-Mode Members, 2025 ▸), *Neovim* (Neovim, 2025 ▸) and *JetBrains* IDEs, *e.g.IntelliJ IDEA* (JetBrains, 2025*a* ▸) and *PyCharm*, usually via LSP plugins. Since any LSP-compliant language tool can be integrated with any LSP-compliant code editor, the CIF language support of the *VS Code* CIF extension can be applied to other code editors and IDEs with relatively little effort. So far, we have prototyped the LSP with *PyCharm*.

## 3. Features

**Figure 1:**
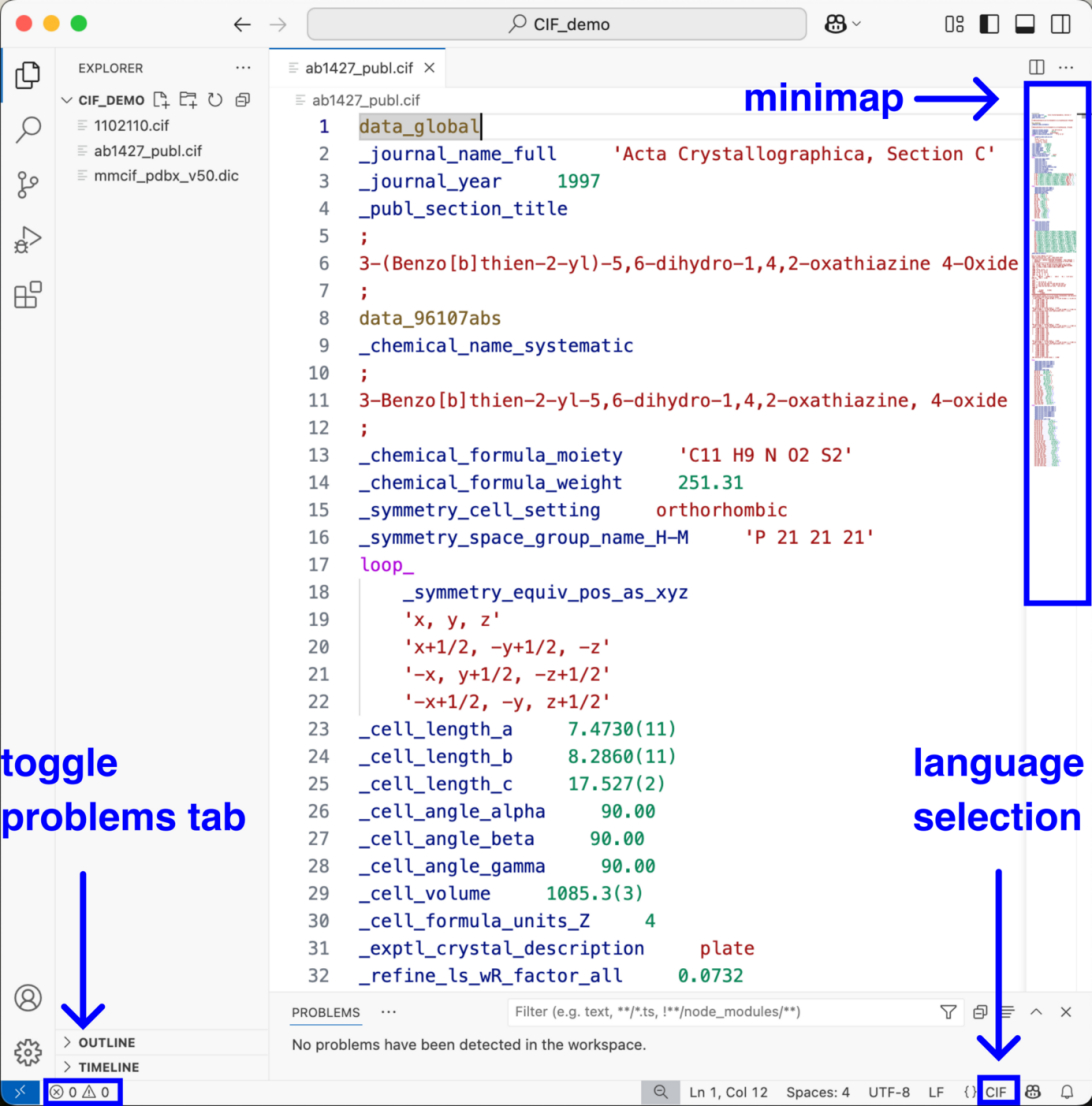
An excerpt of a CIF (Gallagher *et al.*, 1997 ▸) in the *VS Code* editor with the ‘light modern’ colour theme. The minimap of the CIF can be used to scroll the file content. The language selection option on the status bar can be used to select the programming language for dictionary files, which *VS Code* does not recognize as CIF syntax because they have different filename extensions.

Syntax highlighting gives specific colours for different CIF items, while the exact colours depend on the colour theme of the *VS Code* editor. An example of the light modern theme is shown in Fig. 1 ▸. In this case, the block code, *i.e.* the name of the data block, is highlighted in dark yellow, data names in blue, data items (characters or text) in red, numbers in green, loops in magenta, characters ‘?’ (unknown value) and ‘.’ (inapplicable value) in blue, and comment lines in green. The user may choose different visual themes or customize the colour theme of the editor and the syntax highlighting colours will adapt. The setting is located under ‘Code’ > ‘Settings’ > ‘Themes’ > ‘Color Theme’ in a Mac operating system and ‘File’ > ‘Preferences’ > ‘Themes’ > ‘Color Theme’ in a Windows operating system. For example, in the dark modern theme, the block code is highlighted in yellow, data names in light blue, data items (characters or text) in orange, numbers in light green, loops in violet, characters ‘?’ and ‘.’ in dark blue, and comment lines in green. *VS Code* has two built-in high-contrast themes with dark and light backgrounds, and other colour themes that support accessibility, such as Night Owl Black (Johnson, 2019 ▸), can be installed as extensions.

**Figure 2:**
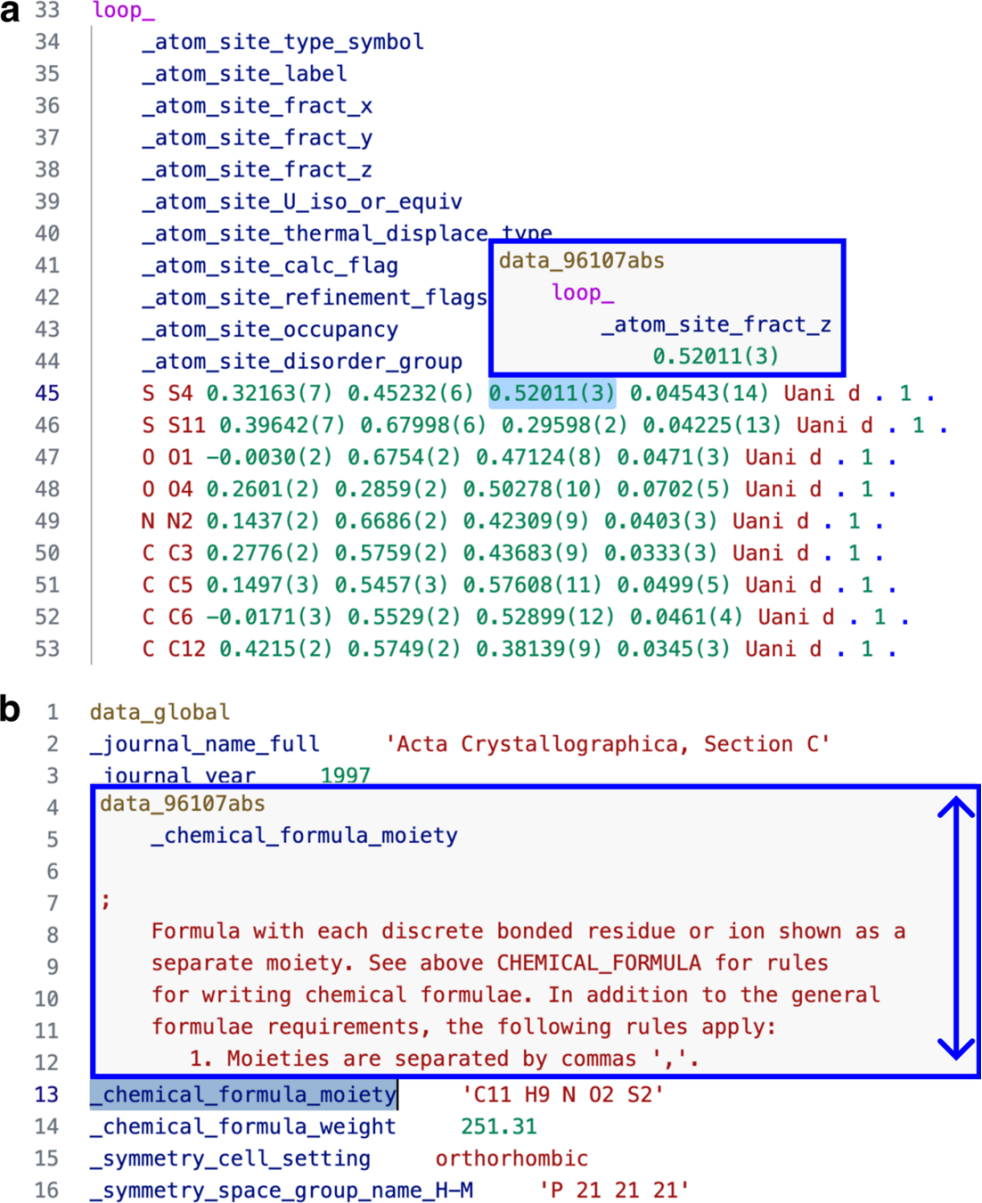
(*a*) Hovering displays the dependencies between the tag–value pairs, loops and data block name using syntax highlighting. (*b*) Hovering over a data name displays the description in the installed CIF dictionaries.

In the *VS Code* CIF extension, hovering over a data name or data item reveals the connection between each data name and data item with indentation that reflects the hierarchical structure of the elements. For example, data block data_96107abs has a loop which contains a data name _atom_site_fract_z with a data value 0.52011(3) [Fig. 2 ▸(*a*)]. This assists the interpretation of data items in loops that contain multiple data names, *i.e.* loops presenting large amounts of tabular data. The syntax highlighting is repeated in the hover window. Data items outside loops show the hierarchy of the data item relative to the data name and the data block name. Hovering over a data name shows the description of the data name in the installed CIF dictionaries [Fig. 2 ▸(*b*)].

**Figure 3:**
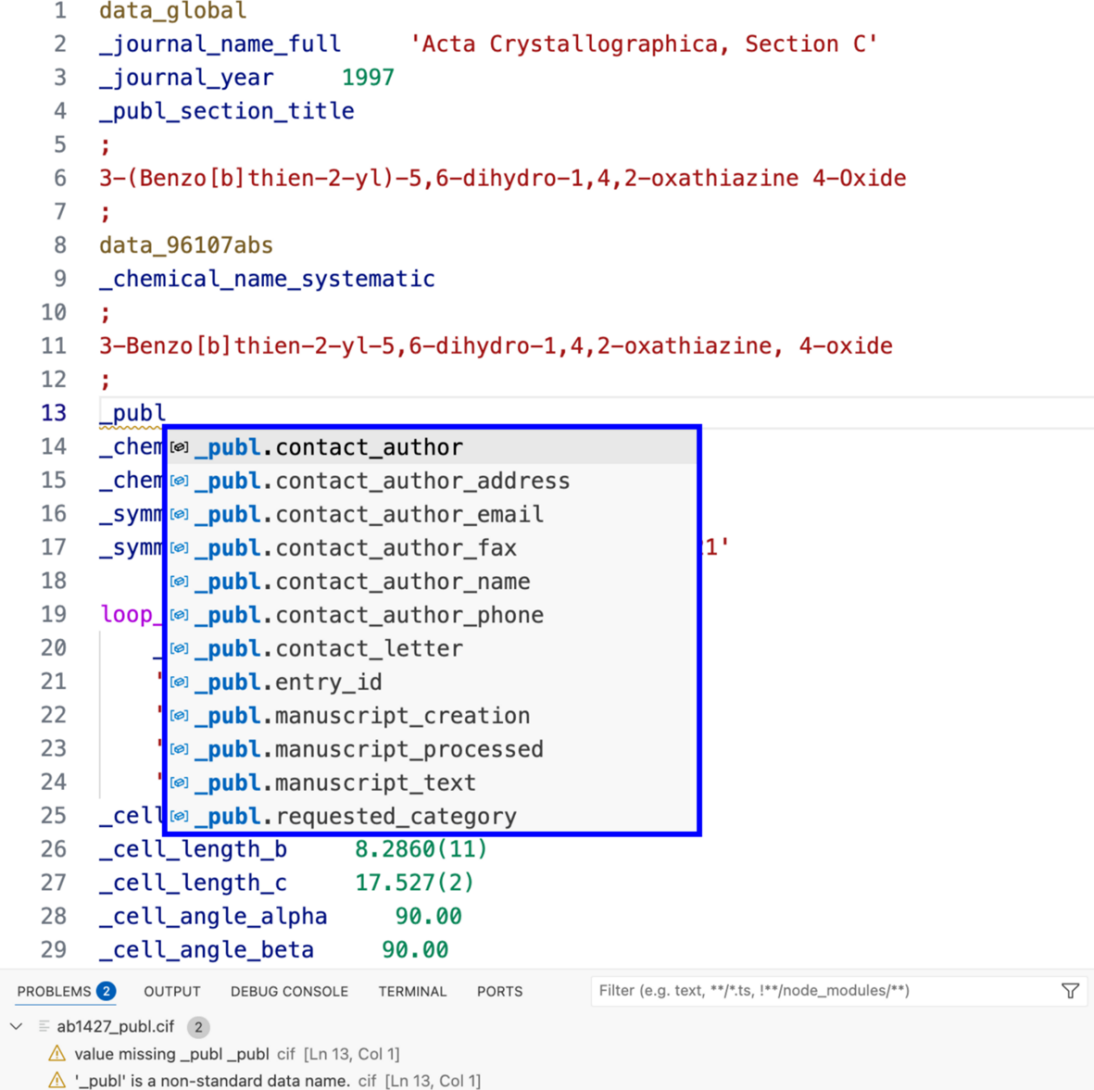
Demonstration of the auto completion feature in the *VS Code* CIF extension.

The installed CIF dictionaries are used for auto completion. While typing a new CIF data name, a list of standard data names defined in the dictionary files are suggested for auto completion. When a data name category is typed, the list shows the options for that category in alphabetical order. In the example in Fig. 3 ▸, typing ‘_publ’ launches a list of standard data name options, which can be scrolled with the arrow buttons or the mouse. Alternatively, typing ‘_author’ shows data names from different categories, including _audit, _publ and _citation. The user only needs to remember part of the data name, either from the category, topic or subtopic.

**Figure 4:**
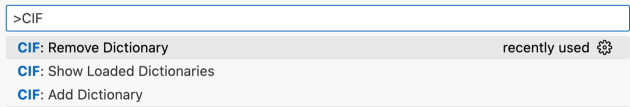
The command palette options for adding, removing and viewing installed CIF dictionaries.

Common syntactic errors in CIFs are duplicated data names in the same data block, missing block codes, errors in loop structures and errors in formatting the data values (Merkys *et al.*, 2016 ▸; Todorov, 2006 ▸). The *VS Code* CIF extension detects these common syntactic errors: an empty file, an empty data block, a missing data block code, a missing data identifier, a duplicate data block name, a duplicate data name, an invalid loop structure, a missing data value after a data name, an unclosed save frame, too long a line above 2048 characters, too long a data name over 80 characters, errors in formatting strings and non-ASCII characters used in CIF 1.1. The syntax error checking is perfomed in real time as the user edits the file. The parser also compares the data names against the included CIF dictionaries to identify non-standard data names, *i.e.* data names that are not defined in the IUCr COMCIFS approved dictionaries (IUCr, 2025*c* ▸). In addition to the default dictionaries, the software has an option to add a new dictionary. This feature is accessed by viewing the command palette (‘View’ > ‘Command Palette’), searching for ‘cif’ and choosing the option ‘CIF: Add dictionary’. Other useful commands are ‘CIF: Show Loaded Dictionaries’ and ‘CIF: Remove Dictionary’, which apply to user-added dictionaries (Fig. 4 ▸). With these options, the user can customize the available dictionaries according to their preferences.

The loaded dictionaries are also used to validate the values of data items for allowed data type, permitted range and correct enumeration, if this information is provided in the dictionaries. Certain data items must have an integer or floating number as a value and the value type is checked by the *VS Code* CIF extension. For example, _chemical_formula_weight must be a real number larger than 1.0 and _symmetry_cell_setting one of the allowed enumerations, such as orthorhombic.

**Figure 5:**
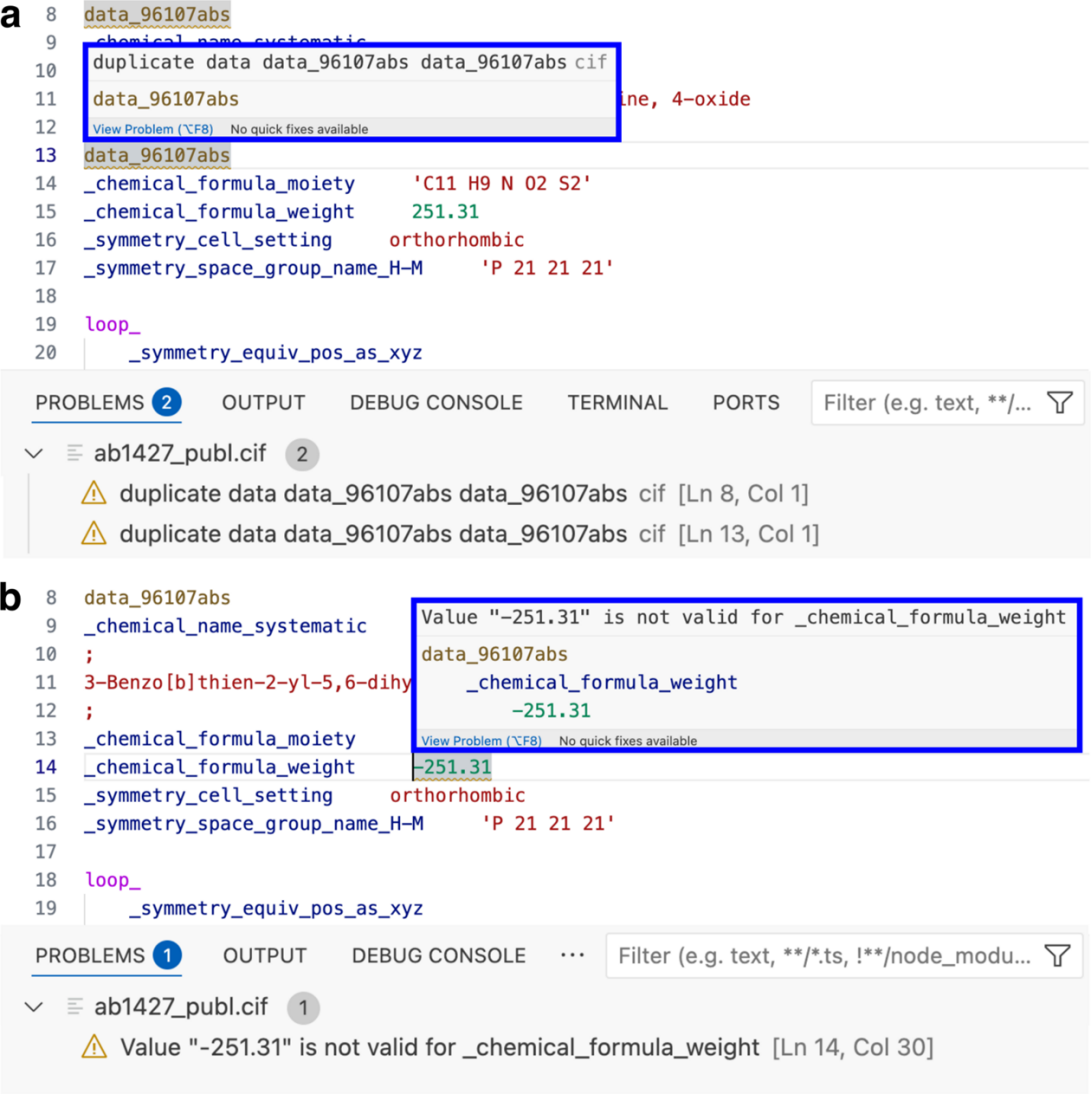
Examples of (*a*) the syntax error checking for duplicate data block names and (*b*) validation of the CIF content. The erroneous construct is underlined with a wavy yellow line and the warning is explained in the hover information and on the ‘PROBLEMS’ tab.

When the parser detects a syntax error or a validation error, the position of the problem is underlined with a dark-yellow wavy line, or with multiple lines in the high-contrast theme. Clicking the status bar opens the bottom panel and the ‘PROBLEMS’ tab, where a warning is shown with a description of the detected error, line number and column position (Fig. 5 ▸). Hovering over the error-giving construct will show the related warning.

It is possible to disable error checking for non-standard data names from the *VS Code* CIF extension settings, for example, if the user does not want warnings for the private data names that often occur in their files. The option is accessed from the gear icon of the *VS Code* CIF extension.

## 4. Conclusion

The *VS Code* CIF extension offers crystallographers an advanced text editor for viewing and manual editing of CIF and dictionary files. The current version available at the *Visual Studio* Marketplace and Github contains all standard CIF dictionaries approved by the IUCr COMCIFS by May 2025, and the user can upload their own dictionaries, such as private dictionaries or macromolecular dictionaries maintained by the Worldwide Protein Data Bank (wwPDB).

The most valuable features of the *VS Code* CIF extension are syntax highlighting, easy access to data name information with the hover function, and validation of the CIF syntax and file information against the CIF dictionaries on the fly. So far, validation of the data value types, ranges and enumerations have been demonstrated and the validation can be improved to cover more physically meaningful values in the future. The validation of the CIF content is more thorough when using, for example, the *checkCIF* publishing service, but the *VS Code* CIF extension offers instant syntax error checking and validation without sending data to an external server, which can be seen as a benefit.

The parser developed for the *VS Code* CIF extension could also be adapted for general purpose use, including integration into web applications and other scientific software beyond the scope of the extension. The development of future *VS Code* CIF extension features will be kept updated in the documentation.

## Figures and Tables

**Table 1:** The token types and regular expressions of the *Regex Table Lexer* in the *VS Code* CIF extension version 1.0.0

CIF example†	Token type	Regular expression CIF 1.1‡	Regular expression CIF 2.0§
_cell_volume	TAG	/^_[^\s]+(?=($|\s))/	
#comment line	COMMENT	/^#.*(?=($|\n))/	
data_96107abs	DATA	/^DATA_[^\s]+(?=($|\s))/i	
loop_	LOOP	/^LOOP_(?=($|\s))/i	
save_	SAVE_END	/^SAVE_(?=($|\s))/i	
save_CIF_CORE	SAVE	/^SAVE_[^\s]+(?=($|\s))/i	
global_	GLOBAL	/^GLOBAL_(?=($|\s))/i	
stop_	STOP	/^STOP_(?=($|\s))/i	
’’’O’Neil’’’	CIF2_TRIPLE		/^’’’(?!’’’).*’’’/
’C11 H9 N O2 S2’	SINGLE	/^’(?:[^’\n]|’(?!\s|$))*’(?!\S)/	/^’[^’\n]*’/
"C11 H9 N O2 S2"	DOUBLE	/^"(?:[^"\n]|"(?!\s|$))*"(?!\S)/	/^"[^"\n]*"/
;	MULTILINE	/^\n;(\n|.)*?\n;/	
sodium diclofenac			
;			
90.00	NUMBER	/^([+-]?(?:\d+(?:\.\d*)?|\.\d+)(?:[eE][+-]?\d+)?)(\(\d+\))?(?=($|[\s]))/	
.	DOT	/^(\.)(?=($|\s))/	
?	QUESTION	/^(\?)(?=($|\s))/	
[	CIF2_LIST_START		/^\[/
]	CIF2_LIST_END		/^\](?=($|\s))/i
{	CIF2_TABLE_START		/^{/
:	CIF2_TABLE_DELIMITER		/^:/
}	CIF2_TABLE_END		/^}//
plate	UNQUOTED	/^[^\s]+/	/^[^\s\]}]+/
	WHITESPACE	/^[^\S\n]+/	
	NEWLINE	/^\n/	

† The CIF language element corresponding to the lexer token.

‡ Regular expressions for checking the CIF 1.1 syntax; an empty cell indicates that the language feature is not used in CIF 1.1.

§ Differences in the regular expressions for CIF 2.0 syntax; an empty cell indicates that the regular expression is the same as in CIF 1.1.

**Table 2:** *TextMate* scopes and regular expressions of the syntax highlighting

Scope name	Match
variable.other.cif	(?:(?<=^)|(?<=\s))_[^\s]+(?=($|\s))
comment.line.number-sign.cif	(?:(?<=^)|(?<=\s))#.*$
entity.name.function.cif	(?:(?<=^)|(?<=\s))(?i)DATA_[^\s]+(?=($|\s))
keyword.control.cif	(?:(?<=^)|(?<=\s))(?i)LOOP_(?=($|\s))
entity.name.function.cif	(?:(?<=^)|(?<=\s))(?i)SAVE_[^\s]*(?=($|\s))
entity.name.function.cif	(?:(?<=^)|(?<=\s))(?i)GLOBAL_(?=($|\s))
entity.name.function.cif	(?:(?<=^)|(?<=\s))(?i)STOP_(?=($|\s))
string.quoted.single.cif	’(?:[^’]|’(?!\s|$))*’(?!\S)
string.quoted.double.cif	\"(?:[^\"]|\"(?!\s|$))*\"(?!\S)
string.quoted.other.cif	begin: ^; end: ^;
constant.numeric.cif	(?:(?<=^)|(?<=\s))([+-]?((\d+(?:\.\d*)?)|(\.\d+))(?:[eE][+-]?\d+)?)(\(\d+\))?(?=($|\s))
constant.language.cif	(?:(?<=^)|(?<=\s))(\.)(?=($|\s))
constant.language.cif	(?:(?<=^)|(?<=\s))(\?)(?=($|\s))
string.unquoted.cif	[^\s]+

## Data Availability

The source code for the *VS Code* CIF extension is available at Github, https://github.com/hmkainul/vscode-cif/.
